# 并发严重凝血障碍的妊娠期急性脂肪肝1例及文献复习

**DOI:** 10.3760/cma.j.cn121090-20241130-00509

**Published:** 2024-12

**Authors:** 新 张, 红彬 孟, 祥林 孟, 林 崔, 荣祎 曹, 丽瑛 蔡

**Affiliations:** 哈尔滨医科大学附属第一医院产科，哈尔滨 150001 Obstetrics Department of the First Affiliated Hospital of Harbin Medical University, Harbin 150001, China

## Abstract

妊娠期急性脂肪肝（AFLP）是产科相对罕见的危急重症，由于其临床表现的非特异性，早期难以确诊。然而早期识别、及时终止妊娠对AFLP的治疗意义重大，对降低死亡率至关重要。本文详细报告1例妊娠37周的AFLP患者，主要表现为恶心呕吐1周，皮肤黄染2 d。因该患者病程相对较长，紧急入院后仍发生了胎死宫内的遗憾结果，并在急诊剖宫取胎术后并发了严重的凝血功能障碍、肺水肿、胰腺炎等多器官功能损伤。产科联合麻醉科、ICU、血液科、输血科等多学科团队，在紧急终止妊娠的同时，积极纠正凝血障碍及多器官损伤，最终患者预后良好。该病例提示我们早期识别对治疗AFLP患者的重要性，严密监测患者凝血功能变化从而指导临床治疗。

## Abstract (en)

Acute fatty liver in pregnancy（Acute fatty liver of pregnancy, AFLP）in pregnancy is a relatively rare critical condition in obstetrics, which is difficult to distinguish in the early stages due to the non-specificity of its clinical manifestations. However, early recognition and timely termination of pregnancy is significant for the treatment of AFLP and is crucial to reduce mortality. This paper will report in detail a patient with AFLP at 37 weeks' gestation, mainly presenting with nausea and vomiting for 1 week and yellow skin for 2 days. Due to the relatively long course of the disease, the patient suffered a regrettable result of fetal death after emergency admission, and severe coagulation dysfunction, pulmonary edema, and pancreatitis occurred. The multidisciplinary team from the department of anesthesiology, ICU, hematology, blood transfusion department and so on, actively corrected coagulation disorders and multiple organ injuries in the emergency termination of pregnancy, and the patient had a good prognosis. The importance of early identification for the treatment of AFLP patients can be demonstrated, and the changes of coagulation function can be closely monitored to guide clinical treatment.

妊娠期急性脂肪肝（Acute fatty liver of pregnancy，AFLP）是一种通常发生在妊娠晚期的并发症，病情凶险，进展迅速，对母儿的生命产生严重威胁。其特征是肝实质脂肪浸润，从而导致急性肝功能障碍，同时可诱发凝血功能障碍、电解质失衡和多器官衰竭[1]。AFLP的临床表现主要为非特异性的消化道症状，如恶心、呕吐、腹痛等，随着病情进展可能出现肝衰竭症状，如黄疸、腹水、凝血障碍等[2]。早期识别、及时终止妊娠可有效阻止病情进展，降低母儿死亡率，但因其临床表现的非特异性，诊断AFLP具有一定的挑战。我们报道1例AFLP病例，为产科医师在诊断及逐步治疗方面提供参考。

## 病例资料

患者，女，26岁，主诉：停经37周，恶心呕吐1周，皮肤黄染2 d。平素月经规律，末次月经时间：2021年8月16日，预产期：2022年5月23日。孕期规律产检，NT、唐筛、糖耐量检查无异常。患者自诉1周前无诱因出现恶心、呕吐，近3 d加重，伴轻微上腹痛，2 d前发现尿色黄及皮肤黄染，就诊于当地医院，建议转至我院。孕1产0，既往史无特殊。查体：神志清楚，体温36.5 °C，呼吸17次/min，心率95次/min，血压135/90 mmHg，复测血压122/84 mmHg。皮肤、巩膜黄染，面部可见散在瘀斑。腹部膨隆，腹软，无压痛、反跳痛。宫高35 cm，腹围105 cm，无宫缩，无阴道流血，无阴道流液，到达产科急诊室时胎心80～120次/min，入院后未闻及胎心，双下肢水肿（+）。辅助检查：产科超声回报：妊娠37周左右活单胎，头位，多次探测胎儿心率持续性缓慢，胎盘老化，羊水少，孕妇宫颈短颈管线尚闭合。急诊室胎心监测提示胎心率基线140次/min，可见频发减速，最低达110次/min，无自身变异性。入院后床旁超声探测无胎心。入院实验室检查：WBC 15.92×10^9^/L，中性粒细胞占83.5％，HGB 157 g/L，PLT 101×10^9^/L；凝血象：PT 13.6 s，APTT 31.9 s，FIB 1.38 g/L，D-二聚体18.07 mg/L。生化系列：ALT 339.3 U/L，AST 218.2 U/L，总胆红素284 µmol/L，总胆汁酸101.5 µmol/L，肌酐179.1 µmol/L，尿酸：699.8 µmol/L，血糖2.59 mmol/L，白蛋白32.3 g/L。尿常规：尿蛋白（++++），尿胆原（+），尿胆红素（+），余化验未见明显异常。

由于患者出现恶心、呕吐、黄疸的消化道症状，加上化验回报提示凝血功能障碍及低血糖，首先考虑为AFLP。依据Swansea标准，符合6条及以上即可明确诊断。该患者符合8条标准，可明确诊断为AFLP。结合其他化验回报，该患者诊断如下：AFLP、妊娠期肝内胆汁淤积症（重度）、妊娠期肝损伤、肾功能不全、妊娠合并凝血功能障碍、妊娠合并低纤维蛋白原血症、黄疸、死胎、妊娠37周，孕1产0，头位。首要措施为尽快终止妊娠。因患者无临产迹象，短时间无法阴道分娩，给予急诊剖宫取胎术。因患者凝血功能障碍，术中出血风险高，给予充分备血、输纤维蛋白原并联合多学科团队进行全程管理。

术中探查见各层组织弥漫性出血不凝，腹膜、子宫膀胱反折黄染，可见弥漫性出血点，子宫表面黄染，胎盘黄染，胎盘剥离后宫腔弥漫性出血，子宫收缩不良，给予子宫加压缝合、卡前列素氨丁三醇注射液250 µg肌注，反复消毒宫腔后给予宫腔填塞长纱条。腹腔留置引流管两枚，腹直肌表面留置引流管一枚。手术时间5月2日15:55–17:30，出血500 ml，尿量100 ml（颜色深黄），输血浆360 ml，输液1 000 ml。术后转入ICU继续治疗。患者于ICU给予机械通气、预防感染、纠正低蛋白、抑酸、保肝、利胆、镇痛镇静及继续输血治疗，同时间隔2 h监测血常规及凝血象。

患者术后凝血障碍不断恶化，凝血象、血常规变化趋势如图1、2所示。术后16 h出现切口瘀斑，术后25 h左右APTT报告危急值，同时血小板下降至（42～47）×10^9^/L，血红蛋白降至70 g/L左右。继续输注红细胞、血浆、纤维蛋白原、血小板，并给予血浆置换及连续性肾脏替代治疗（CRRT）。术后35 h患者腹壁引流持续缓慢增多并可见引流口持续渗血，自270 ml增多至560 ml，急查床旁超声：左下腹直肌70.2 mm×20.6 mm低回声；右下腹直肌29 mm×11.9 mm低回声。考虑腹直肌活动性出血，向患者家属交代病情后要求再次手术治疗。

**图1:**
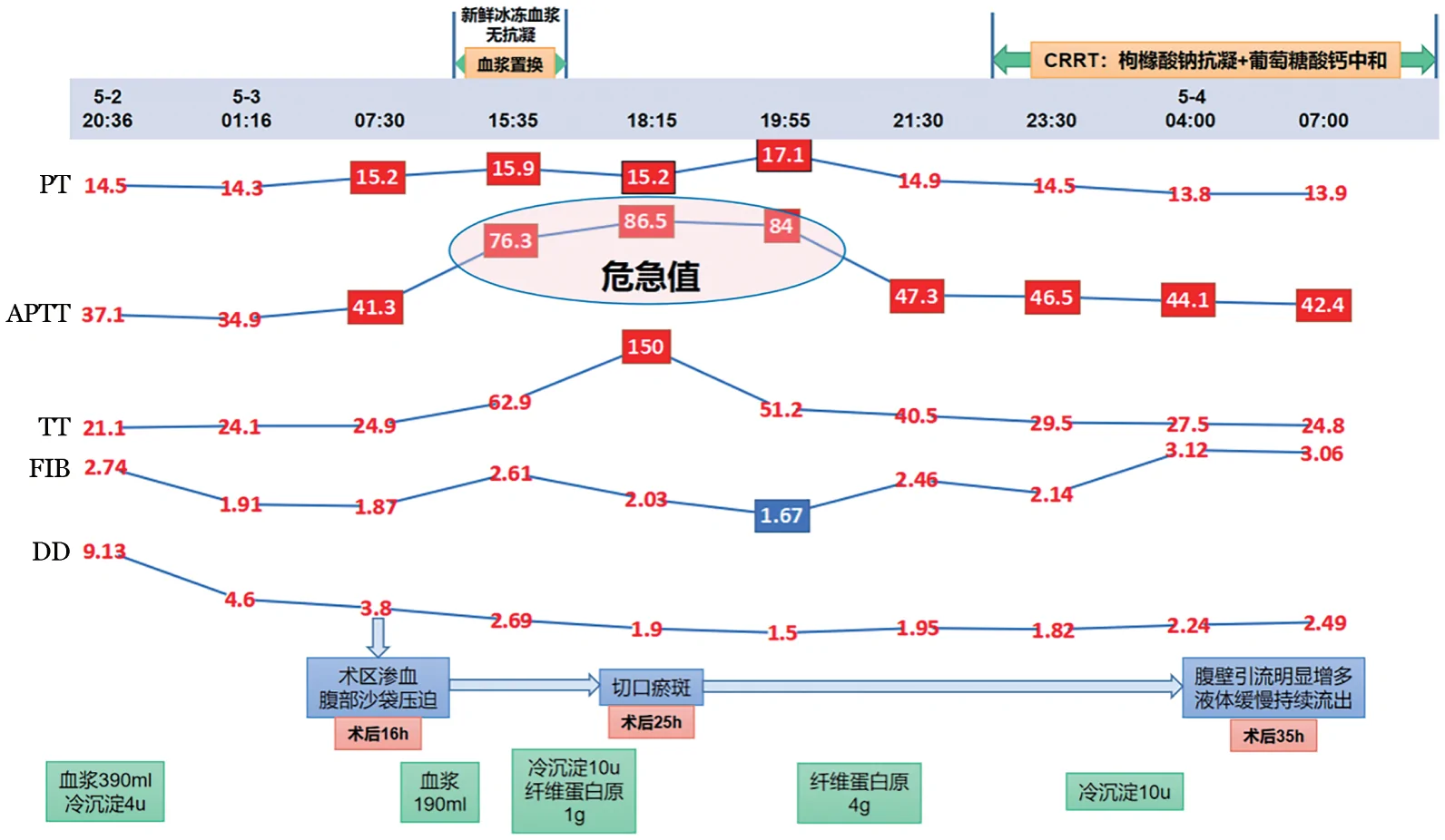
剖宫取胎术后凝血象变化（上方为化验回报时间）

**图2:**
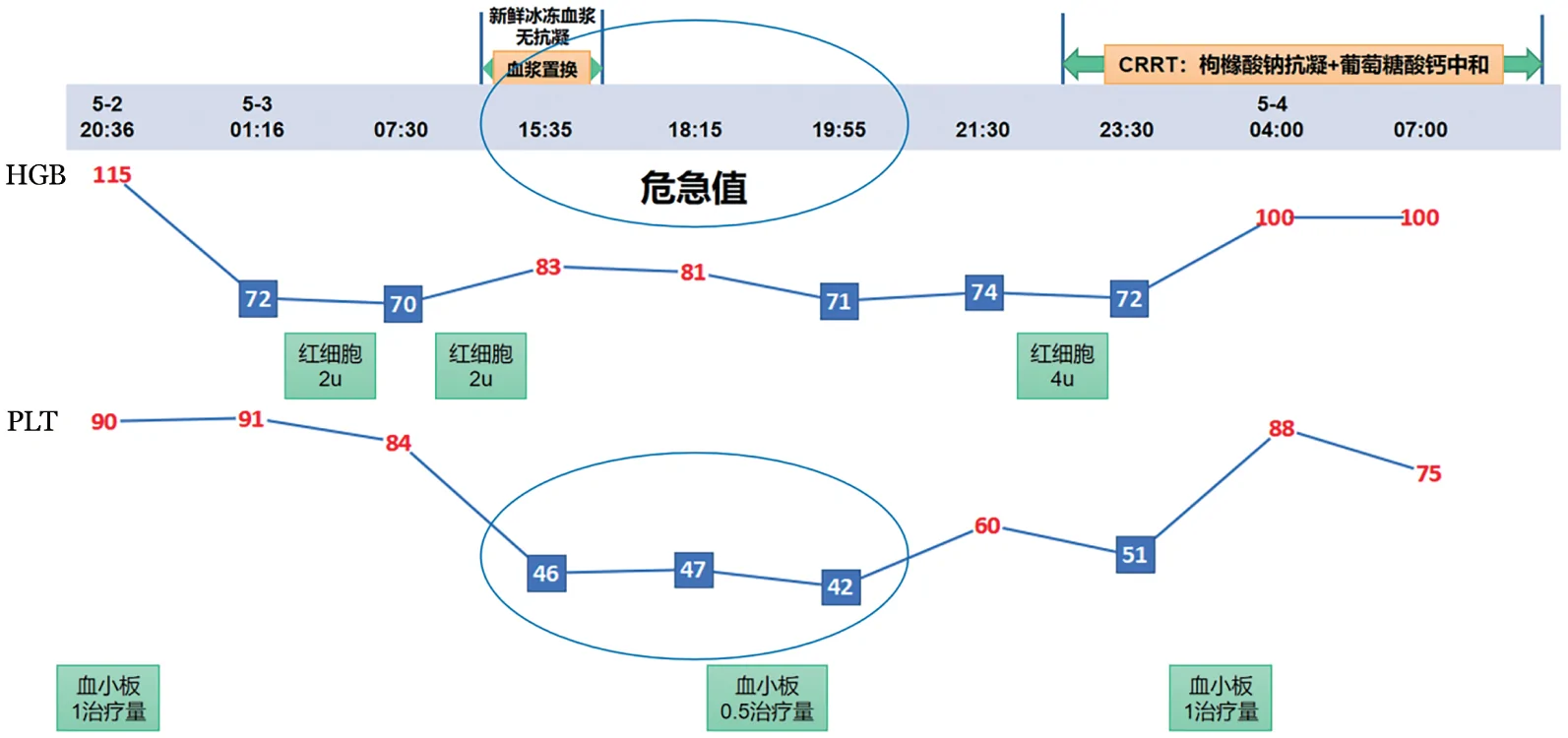
剖宫取胎术后血常规变化（上方为化验回报时间）

再次手术术中腹直肌表面可见大小约6 cm×5 cm血凝块，腹直肌表面及腹直肌下方未见活动性出血点，用温生理盐水反复冲洗切口后，腹直肌表面少量渗血、电凝处理，腹直肌表面放置止血纱布。腹直肌前方留置引流管一枚。术中出血50 ml，输血浆600 ml，冷沉淀20 U。术后转入ICU继续输注血制品及凝血物质纠正凝血功能，并继续间隔2 h监测血常规及凝血象。

再次手术后凝血障碍仍然存在，给予血液科急会诊，遵会诊急查内外源性凝血因子，输注凝血酶原复合物，并继续血浆置换及CRRT，之后凝血象回报趋于稳定（图3）。因患者血小板水平仍然较低［（50～60）×10^9^/L］（图4）、凝血障碍持续存在，因此延迟取出宫腔填塞纱布（剖宫取胎术后72 h），并在取出纱布前输血小板以降低再次出血概率。

**图3:**
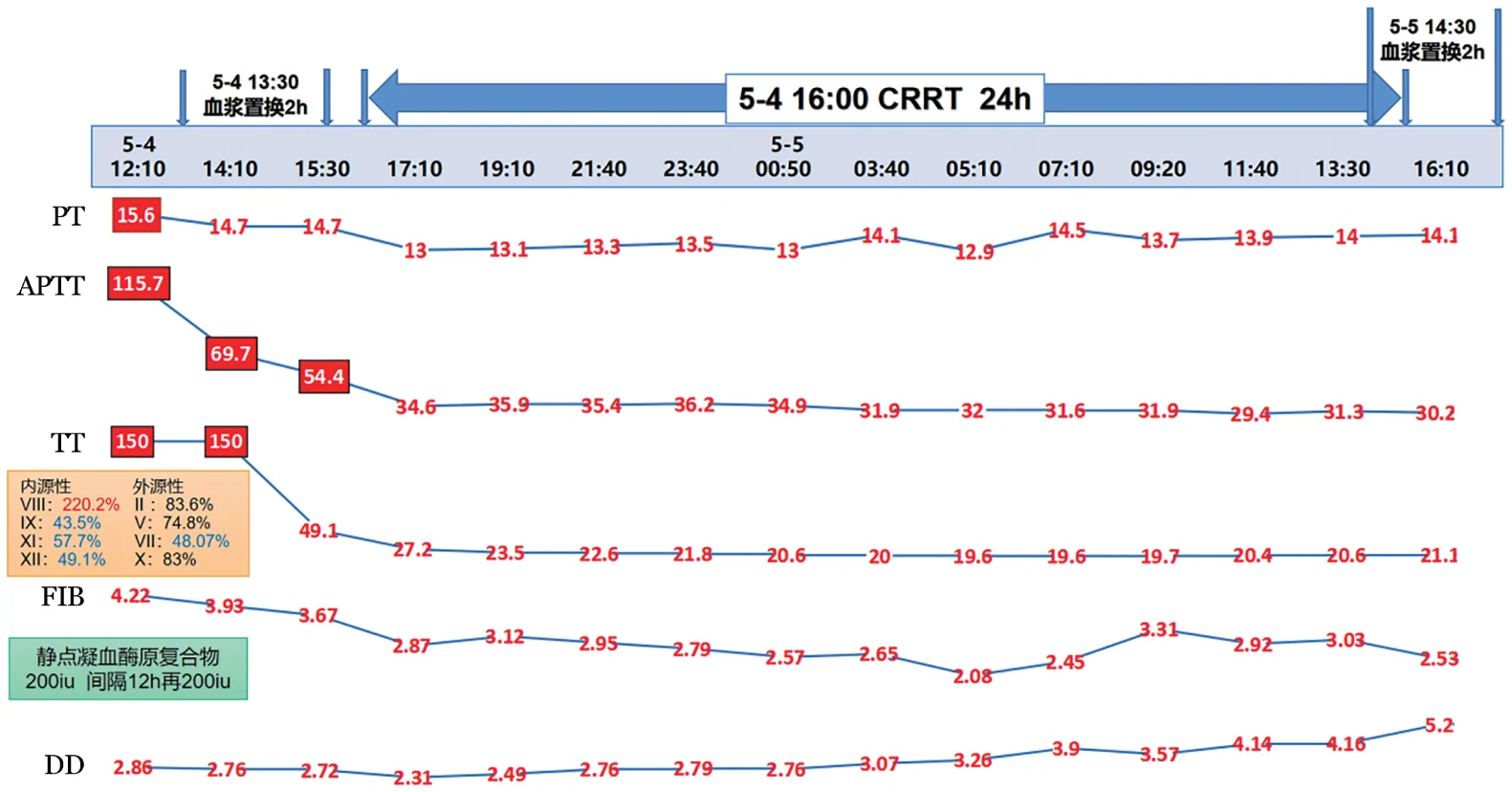
再次手术后凝血象回报（上方为实验室检查回报时间）

**图4:**
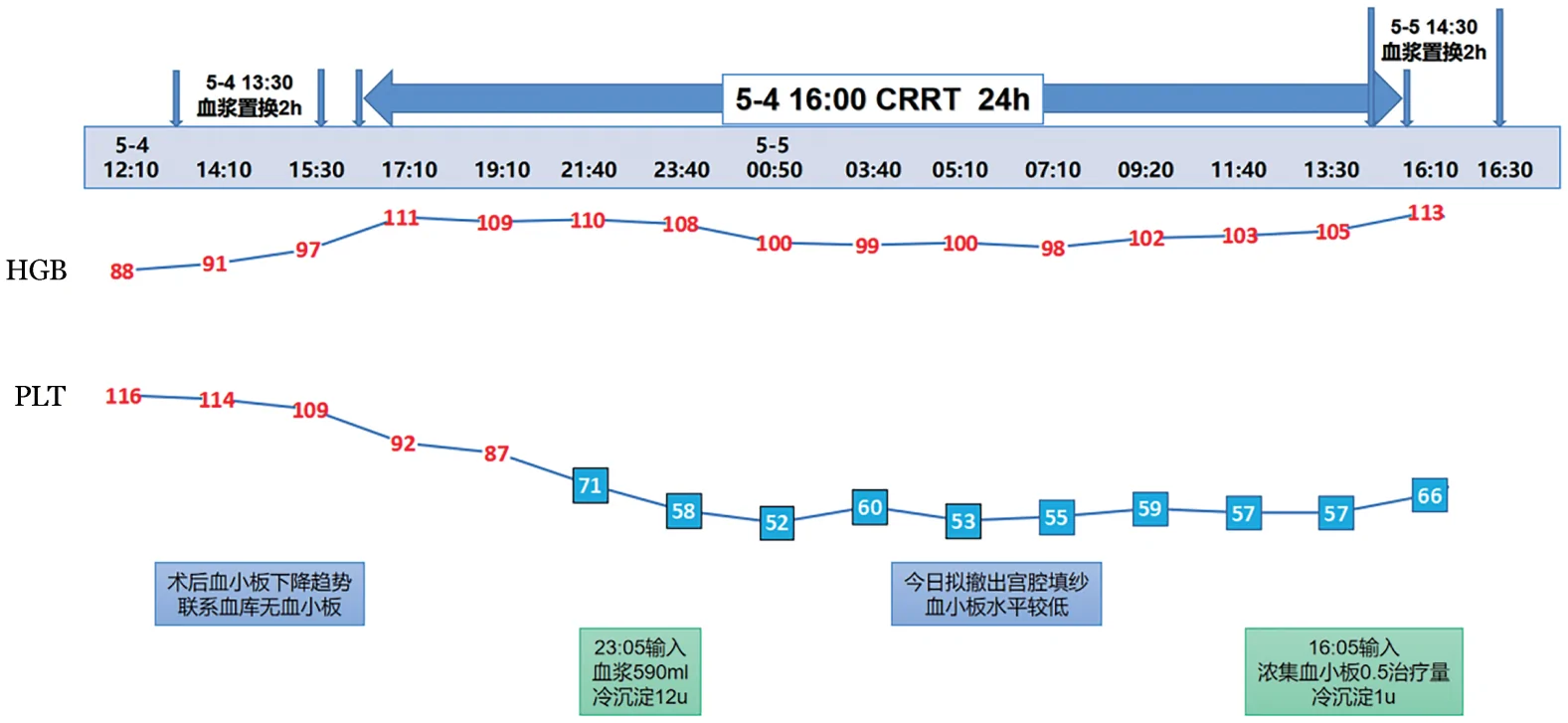
再次手术后血常规回报（上方为实验室检查回报时间）

缓慢取出宫腔纱布后25 min出血量约10 ml，给予卡前列素氨丁三醇注射液250 µg肌注，观察43 min清除宫腔积血约200 ml，再观察3 min再次清除宫腔积血200 ml，出血汹涌。因患者此时血小板水平低、凝血功能尚未完全稳定，若继续观察可能导致出血量进一步增多、凝血功能再度崩溃，立即给予宫腔止血球囊压迫止血。止血成功后继续监测血常规及凝血象变化。止血球囊放置24 h后给予缓慢抽出囊内液体（间隔1 h抽取50 ml），待全部液体抽净后，成功撤出宫腔止血球囊，同时血常规及凝血象回报结果趋于稳定。肝肾功能、胆汁酸、胆红素也逐步恢复（表1）。剖宫取胎术后第5日拔除腹壁引流管，术后第7日脱离呼吸机，术后第8日拔除腹腔引流管，术后第9日转回产科病房，在治疗胰腺炎、肝功能异常、反复发热后，患者于剖宫取胎术后第19日出院。患者于住院期间共输注血浆2 910 ml、红细胞16 U、冷沉淀77 U、血小板7.5治疗量、纤维蛋白原11 g、凝血酶原复合物400 IU。

**表1:** 患者术后肝肾功能检测结果

日期	ALT（U/L）	AST（U/L）	总胆红素（µmol/L）	总胆汁酸（µmol/L）	尿素（mmol/L）	肌酐（µmol/L）	尿酸（µmol/L）	钙（mmol/L）	磷（mmol/L）	镁（mmol/L）	钾（mmol/L）	钠（mmol/L）	氯（mmol/L）
5月3日	96.1	61.8	222.5	93.1	9.65	216.5	663.8	2.36	1.49	1.17	3.96	135.9	98.3
5月4日	34.9	66.6	194.4	124.3	9.46	220	374.2	2.65	1.05	0.87	4.16	141.5	100.9
5月5日	19.7	31.9	140.2	126.3	6.15	115.6	184.9	3.31	0.92	0.92	3.14	138	100.6
5月6日	15.5	18.8	141.4	130.6	7.39	140.6	269.8	2.86	0.85	0.75	4.79	143.5	105.4
5月7日	16.7	22.9	154.1	167.1	13.81	160.3	356.9	2.38	0.79	0.63	3.65	145.6	105.4
5月8日	19.5	23.4	175.6	98.6	19.75	133.7	339.3	2.1	0.57	0.69	3.32	147.6	106.6
5月9日	25.5	33.4	106.1	128.1	17.61	87.1	304.2	1.93	0.99	0.73	3.91	153.3	116.2
5月10日	48.2	49.2	70.7	53.8	13.85	65.6	225.5	2.12	0.43	0.88	4.39	152.1	116.9
5月11日	80.4	45.5	61.6	45.6	11.92	48.7	224.1	2.05	1.11	0.95	4.43	146.1	113.7
5月12日	98.9	56.3	71.8	12.2	9.58	55	213.6	1.96	0.98	0.92	3.62	141.7	111.6
5月14日	98.2	68.1	62.9	8.2	6.83	48.3	209.3	2.01	0.96	0.94	3.67	134.6	102.8
5月16日	97.3	63.8	48.1	5.5	6.51	50	272.3	2.1	1.19	0.93	3.94	135.8	103
5月18日	85.6	64.5	37.1	9	–	–	–	–	–	–	–	–	–
5月21日	68.1	63.8	24.2	17.5	2.86	41.7	249.3	2.12	1.28	0.86	4.16	139.8	107.1

参考范围	5～35	8～40	3.4～21	0.01～10	2.9～8.2	35～80	142～339	2.11～2.52	0.85～1.51	0.75～1.02	3.5～5.3	137～147	99～110

患者出院后1周复查结果：WBC 5.22×10^9^/L，HGB 130 g/L，PLT 267×10^9^/L，ALT 51.1 U/L，AST 53.6 U/L。凝血象正常范围内。肝胆胰脾超声：轻度脂肪肝，胆囊炎。妇科超声：宫腔内混合回声（积血可能），子宫前壁峡部回声不均，宫颈回声欠均。患者出院后9个月后再次妊娠，因稽留流产于我科行超声引导下清宫术。

## 讨论及文献复习

AFLP为产科特有的危急重症，发病率较低且病程进展迅速，若不及时干预可导致严重的母婴结局。AFLP通常发生于妊娠30～38周，由于30周后母亲脂肪分解增加，更多的脂肪酸通过胎盘转移到胎儿，触发胎盘和胎儿中的脂质代谢和线粒体脂肪酸氧化增加，从而导致AFLP的发生[3]。该病的高危因素包括：AFLP病史、HELLP综合征（hemolgsis，elevated liver function and low platelet count syndrome）、子痫前期、妊娠期肝内胆汁淤积症（Intrahepatic cholestasis in pregnancy，ICP）、男性胎儿及多胎妊娠[4]–[5]，该患者的高危因素包括男性胎儿和ICP。该病的临床表现为非特异性，如恶心呕吐、上腹痛等消化道症状，逐步可进展为肝肾功能衰竭等多器官功能障碍，部分患者还可伴随子痫前期，其中20％患者可并发HELLP综合征[6]，因此AFLP与HELLP综合征鉴别较为困难。HELLP综合征为子痫前期的一种严重表型，其组织学检查特点为门静脉周围出血和纤维蛋白沉积，而AFLP的特征是肝脏微泡脂肪浸润。HELLP综合征相比于AFLP表现为更明显的肝损伤，Casey等[7]发现HELLP组的中位AST水平为642（48～7194）IU/L，而AFLP组为85（45～165）IU/L，除此之外HELLP综合征还伴随溶血、血小板减少、水肿、头痛等高血压表现。AFLP则更倾向多器官功能损伤，AFLP患者的肝细胞内脂质进行性积累，使肝细胞脂肪含量增加到13％～19％（正常脂肪含量约为5％），肝糖原分解受损可导致低血糖，胆红素清除障碍导致高胆红素血症及黄疸，肾脏脂肪酸氧化缺陷和肾小管脂肪变性从而表现肾损伤，纤维蛋白原和其他凝血物质生成减少是凝血功能障碍的主要原因[8]–[9]。病毒性肝炎可由肝炎病毒阴性区分，ICP主要表现皮肤瘙痒，消化道症状较轻。

AFLP是一种由Swansea标准支持的排除性诊断，但有研究表明，胃肠道症状+转氨酶+胆汁酸+APTT/PT+胆红素的组合在AFLP的诊断中敏感性为97.6％，特异性为97.1％，而更多的标志物不一定有助于鉴别诊断[10]。该患者入院时化验回报出现白细胞升高、转氨酶、胆红素、胆汁酸、肌酐、尿酸均明显升高，随机血糖明显降低、无出血倾向前提下凝血功能障碍、孕期否认高血压病史，肝炎病毒阴性，且符合8条Swansea标准，可明确诊断。诊断后立即给予剖宫取胎术，因术中凝血障碍广泛渗血，给予充分止血、宫腔填纱，并留置引流管以便观察术后出血情况。与此同时，术前术中术后不断输注纤维蛋白原、血浆、红细胞、冷沉淀、血小板等血制品纠正凝血功能，研究表明应维持纤维蛋白原水平至少为1.5 g/L，并输注血小板以将血小板计数保持在50×10^9^/L以上，并且给予重组因子Ⅶ A（rFⅦ a）可能会减少血制品用量并降低术中切子宫的风险[11]。根据AFLP临床管理指南[12]，该患者病程超过1周，肌酐>133 µmol/L，属极高危患者人群，术后需转入ICU进行重症监护。并于术后间隔2 h复查血常规、凝血象以便及时监测患者凝血功能变化，每日复查肝肾功。该患者于剖宫取胎术后16 h出现切口瘀斑、活动性渗血、腹直肌血肿，考虑与现有研究所表明的部分患者于终止妊娠后出现凝血功能障碍、肝肾功的初始恶化相一致[13]。

患者术后凝血障碍持续存在，延迟取出宫腔纱布，针对晚期产后出血立即宫腔止血球囊压迫止血。对于AFLP合并产后出血的患者，即使患者伴有肝功能衰竭和凝血功能障碍，宫内球囊填塞对控制产后出血的作用也是明确的[14]。由于持续大量输注血制品为人工肝治疗的指征[12]，术后21 h开始血浆置换和CRRT，血浆置换无抗凝，CRRT采取枸橼酸钠抗凝+葡萄糖酸钙中和以减少该治疗对凝血功能的影响。血浆置换可以去除循环内毒素、补充凝血因子和蛋白质，CRRT可以滤除体内多余液体和废物以治疗急性肾损伤[15]，血浆置换联合CRRT可改善患者临床症状、尽快纠正实验室指标异常、缩短住院时间、降低死亡率[16]–[17]。该患者出现了肺水肿、急性胰腺炎和感染的并发症，其中胰腺炎被认为是潜在致命并发症，脂肪酸代谢产物对胰腺的毒性作用可能是并发胰腺炎的机制之一[18]，诊断AFLP的患者都应进行胰腺炎指标的常规筛查[19]。

回顾该患者整个病程，入院1周前即出现恶心呕吐，并未及时就诊，在询问产检医师后未给予特殊处置，后出现症状加重及上腹痛才就诊于当地医院，错失最好的治疗时机，以至于进展到严重且顽固的凝血障碍以及死胎的遗憾结果。因此日常的基础母婴保健教育、提高患者的重视程度及提高基层医师的职业敏感性尤为重要。综上所述，早期识别、及时治疗是改善AFLP患者母婴结局的重中之重，同样离不开多学科团队的相互协作。值得注意的是，此类患者在将来妊娠中应高度警惕再次出现AFLP的可能[20]。

## References

[1] Hadi Y, Kupec J (2024). Fatty Liver in Pregnancy(2023-07-4)[M/OL].

[2] Westbrook RH, Dusheiko G, Williamson C (2016). Pregnancy and liver disease[J]. J Hepatol.

[3] Wong M, Hills F, Vogler K (2020). Acute Fatty Liver of Pregnancy From 18 Weeks' Gestation[J]. Hepatology.

[4] Bacq Y (2011). Liver diseases unique to pregnancy: a 2010 update[J]. Clin Res Hepatol Gastroenterol.

[5] Tran TT, Ahn J, Reau NS (2016). ACG Clinical Guideline: Liver Disease and Pregnancy[J]. Am J Gastroenterol.

[6] Liu J, Ghaziani TT, Wolf JL (2017). Acute Fatty Liver Disease of Pregnancy: Updates in Pathogenesis, Diagnosis, and Management[J]. Am J Gastroenterol.

[7] Casey LC, Fontana RJ, Aday A (2020). Acute Liver Failure (ALF) in Pregnancy: How Much Is Pregnancy Related?[J]. Hepatology.

[8] Nelson DB, Yost NP, Cunningham FG (2013). Acute fatty liver of pregnancy: clinical outcomes and expected duration of recovery[J]. Am J Obstet Gynecol.

[9] Ang SX, Chen CP, Sun FJ (2021). Comparison of maternal and neonatal outcomes between acute fatty liver of pregnancy and hemolysis, elevated liver enzymes and low platelets syndrome: a retrospective cohort study[J]. BMC Pregnancy Childbirth.

[10] Zhong Y, Zhu F, Ding Y (2020). Early diagnostic test for acute fatty liver of pregnancy: a retrospective case control study[J]. BMC Pregnancy Childbirth.

[11] Singh S, Menon A, Thareja S (2011). Recombinant activated factor VIIa in a case of pregnancy with acute hepatic failure and massive blood loss[J]. Med J Armed Forces India.

[12] 中华医学会妇产科学分会产科学组 (2022). 妊娠期急性脂肪肝临床管理指南(2022)[J]. 临床肝胆病杂志.

[13] Patidar R, Gowdra Revannasiddappa K, Ghazanfer M (2024). Key Components of Successful Management of Acute Fatty Liver of Pregnancy: A Case Report and Literature Review[J]. Cureus.

[14] Nelson DB, Byrne JJ, Cunningham FG (2020). Acute Fatty Liver of Pregnancy[J]. Clin Obstet Gynecol.

[15] Siwatch S, De A, Kaur B (2024). Safety and efficacy of plasmapheresis in treatment of acute fatty liver of pregnancy-a systematic review and meta-analysis[J]. Front Med (Lausanne).

[16] Yu CB, Chen JJ, Du WB (2014). Effects of plasma exchange combined with continuous renal replacement therapy on acute fatty liver of pregnancy[J]. Hepatobiliary Pancreat Dis Int.

[17] Kumar A, Sharma A, Mohan Y (2021). Therapeutic plasma exchange in acute fatty liver of pregnancy: a case report and literature review[J]. Pan Afr Med J.

[18] Ye R, Mai Z, Pan X (2021). Acute fatty liver of pregnancy causes severe acute pancreatitis and stillborn fetus: A case report[J]. Medicine (Baltimore).

[19] Moldenhauer JS, O'brien JM, Barton JR (2004). Acute fatty liver of pregnancy associated with pancreatitis: a life-threatening complication[J]. Am J Obstet Gynecol.

[20] Bacq Y, Assor P, Gendrot C (2007). Recurrent acute fatty liver of pregnancy[J]. Gastroenterol Clin Biol.

